# Seropositivity rates for agents of canine vector-borne diseases in Spain: a multicentre study

**DOI:** 10.1186/1756-3305-6-117

**Published:** 2013-04-22

**Authors:** Guadalupe Miró, Ana Montoya, Xavier Roura, Rosa Gálvez, Angel Sainz

**Affiliations:** 1 Department of Animal Health, Veterinary Faculty, Universidad Complutense de Madrid, Madrid, Spain; 2 Hospital Clínic Veterinari, Universitat Autònoma de Barcelona, Barcelona, Spain; 3 Department of Animal Medicine and Surgery, Veterinary Faculty, Universidad Complutense de Madrid, Madrid, Spain

**Keywords:** Leishmaniosis, Heartworm, Ehrlichiosis, Anaplasmosis, Lyme disease, Dog

## Abstract

**Background:**

Controlling canine vector-borne diseases (CVBD) is a major concern, since some of these diseases are serious zoonoses. This study was designed to determine seropositivity rates in Spain for agents causing the following five CVBD: leishmaniosis (*Leishmania infantum*: Li), heartworm (*Dirofilaria immitis*: Di), ehrlichiosis (*Ehrlichia canis*: Ec), anaplasmosis (*Anaplasma phagocytophilum/Anaplasma platys*: An) and Lyme disease (*Borrelia burgdorferi*: Bb).

**Methods:**

Anti-An, -Bb, and -Ec antibodies and the Di antigen were determined using the 4DX SNAP® Test (IDEXX Laboratories) and anti-*L. infantum* (Li) antibodies using the *Leishmania* SNAP® Test (IDEXX Laboratories) in blood and/or serum samples.

**Results:**

Among 1100 dogs examined, overall seropositivity rates were: Li (15.7%), Ec (5%), An (3.1%), Di (1.25%) and Bb (0.4%). While seropositivity towards Bb and Di was similar in all geographic regions, rates were significantly higher in the east of Spain (8.3%) for An, significantly higher in the north (20%) for Ec, and significantly higher in the Southeast (46.6%) and South (27.4%), and significantly lower in the north (0%) for Li.

No statistical associations were observed between sex and the CVBD analyzed (*p ≥ 0.05*) while the following associations with other variables were detected: a higher seropositivity to Ec (40%) and Bb (6.7%) in dogs under one year of age compared with adults (*p < 0.05*); and a higher seropositivity to An and Li in dogs that lived outdoors *versus* indoors (*p = 0.01*; *p < 0.001*, respectively). Seropositivity rates of 2.1%, 0%, 1.7%, 0.5% and 4.2% were recorded respectively for An, Bb, Ec, Di and Li in dogs with no clinical signs (n = 556) versus 3.8%, 0.6%, 7.5%, 1.8% and 25.9% for those with signs (n = 507) suggestive of a CVBD.

**Conclusion:**

The data obtained indicate a risk for dogs in Spain of acquiring any of the five CVBD examined. Veterinarians in the different regions should include these diseases in their differential diagnoses and recommend the use of repellents and other prophylactic measures to prevent disease transmission by arthropod vectors. Public health authorities also need to become more involved in the problem, since some of the CVBD examined here also affect humans.

## Background

The term canine vector-borne diseases (CVBD) includes a wide variety of diseases of infectious or parasitic aetiology whose agents are transmitted by ectoparasites such as ticks, fleas, lice, mosquitoes and sand flies [1]. Controlling these infectious agents is important because some are responsible for serious diseases in humans (e.g. *Anaplasma phagocytophilum, Bartonella* spp*., Borrelia burgdorferi, Leishmania infantum, Thelazia callipaeda*, etc.) [2]. However, their control can be extremely complex since they show a wide geographical distribution, and clinical signs in infected dogs can vary significantly [3,4].

In addition, there is evidence to suggest that changing factors linked to climate and the environment could determine the expansion of the current geographical distribution ranges of these diseases and their arthropod vectors [5-9]. The transport of infected dogs from endemic areas has also been attributed an important role in the spread of CVBD to the north of Europe [10,11]. Owing to an increase in this transport due to new habits such as travelling with dogs or adopting animals from other countries, the epidemiological status of these diseases in Europe, and especially across the Iberian Peninsula including Portugal, has changed considerably [12-14].

CVBD may show no specific clinical signs or clinical-pathological abnormalities, or alternatively may present a varied clinical picture making the diagnosis of a CVBD extremely complex. Animals with subclinical infection have been described to show an increased risk of disease transmission [15,16].

*Anaplasma platys* and *A. phagocytophilum* (An) are the aetiological agents of anaplasmosis, which affects a wide range of vertebrate hosts (rodents, dogs, humans). *A. phagocytophilum* is transmitted by ticks of the genus *Ixodes* and *A. platys* by the tick *Rhipicephalus sanguineus*. Both pathogens infect dogs in which the clinical picture ranges from subclinical disease to acute illness [17]. *A. phagocytophilum* can also infect humans causing febrile syndrome [18]. The reported seroprevalence of *Anaplasma* spp. in Spain has ranged from 5 to 19% for Galicia, Catalonia, Balearic Islands and Castilla-León [19-21].

Lyme disease is an infectious disease caused by spirochetes belonging to the *Borrelia burgdorferi* (Bb) *sensu lato* complex, transmitted by ticks of the genus *Ixodes*. Lyme disease shows a worldwide distribution, although its incidence is increasing in North America and Europe because of its association with this vector [22-24]. Bb affects a wide range of hosts, mainly humans and dogs. In humans, Lyme disease can produce chronic weakness with nonspecific clinical signs (fever, muscle and joint pain). Though few dogs show clinical signs, most are subclinical reservoirs [25,26] and can be used as sentinels for this infection. In Spain, dogs seropositive for *B. burgdorferi* have been detected in Galicia (6.3%) [20], Mallorca (1.3%) [19] and Castilla -León (2.1 to 21%) [27-29].

*Ehrlichia canis* (Ec), an intracellular Gram-negative bacterium that infects monocytes, is the causative agent of canine monocytic ehrlichiosis, and is transmitted by the tick *Rhipicephalus sanguineus*[30]. The disease is characterized by three stages of varying severity. The acute stage produces clinical signs such as apathy, depression, anorexia, dyspnoea, fever, lymphadenopathy, splenomegaly, petechiae and echymotic haemorrhage in the skin and the mucous membranes, epistaxis, and vomiting. Laboratory abnormalities are usually thrombocytopenia, leucopenia and mild to moderate normocytic, normochromic and non-regenerative anaemia. The second stage is subclinical with clinical-pathological abnormalities such as thrombocytopenia, anaemia or hyperproteinemia. The third or chronic stage is characterized by a very complex clinical picture: haemorrhage, weakness, apathy, sustained weight loss, fever, lymphadenopathy, splenomegaly and peripheral oedema in the hind limbs and scrotum and a wide variety of clinical-pathological abnormalities [31,32]. In Spain, the seroprevalence of Ec in dogs ranges from 3.1 to 19%, with cited rates of 3.1-6.5% for Galicia, Madrid and Zaragoza [20,33,34] and higher rates for Mediterranean regions (Catalonia, Valencia, Baleares) [19,35,36] and Castilla-León (12-20%) [35].

*Dirofilaria immitis* (Di) is a filarial worm transmitted by mosquitoes (Culicidae) to carnivores and other hosts. Since the vector is not very host specific, many mammals can become infected including humans [37]. *D. immitis* is a cosmopolitan parasite, mainly found in southern European countries including Spain, where it is endemic in the regions Valencia, Balearic Islands, Andalucia, Aragon and the Canary Islands with prevalences of 6.3-67.02% [38-42]. Lower prevalences have been reported for other regions, although recently cases have been detected in two northern provinces: La Rioja (12%) and La Coruña (4.2%) [43,44]. *D. immitis,* also known as heartworm, mainly affects dogs but has also been detected in cats [45]. In dogs, the course of disease is chronic due to changes in the pulmonary arteries and lung parenchyma [46]. In humans, the parasite cannot complete its whole life cycle, yet produces a serious infection in which parasitic granulomas can be observed in the lung parenchyma [47].

Canine leishmaniosis (CanL), a zoonotic disease endemic in southern Europe caused by the protozoan *Leishmania infantum* (Li), is transmitted to humans and animals by blood-sucking phlebotomine sand flies [48,49]. Until recently, CanL was considered to be limited to the Mediterranean basin, with an estimated seroprevalence in Spain ranging from 3% in the north [50] to 34.6% for Malaga province on the south coast [51]. Northern Spain was considered a non-endemic area but CanL and its sand fly vectors have been detected in the northeast and northwest where the disease was previously unknown [50,52-54]. At our latitude, dogs are considered the main reservoir for human infection [55,56]. Clinical CanL shows a wide spectrum of clinical signs and severity because of the many pathogenic mechanisms involved and the particular immune response produced in the host [57]. The main clinical signs of CanL are one or more of the following: weight loss, lethargy, muscular atrophy, anaemia, lymphadenomegaly, splenomegaly, epistaxis, diarrhoea, renal disorders, ocular lesions, polyarthritis, onycogryphosis and skin lesions [58-60]. In endemic areas, a high proportion of clinically healthy dogs are able to transmit the infection causing a serious public health problem [61]. In Europe, human infection with *L. infantum* is observed mainly in children and immunocompromised adults, but a recent outbreak (2010) in southwest Madrid (Spain) indicates the epidemiology of this disease is complex and subject to constant change [62,63].

Information emerging from Spain on some of these vector borne diseases has been limited. Most studies have addressed canine leishmaniosis in the Mediterranean basin and a few reports have dealt with ehrlichiosis, dirofilariosis, *Borrelia* and *Anaplasma* infection in specific areas of the country.

The present study was designed to establish seropositivity rates and epidemiological associations for these five CVBD by determining antibodies against *Anaplasma* spp*.* (An), *Borrelia burgdorferi* (Bb), *Ehrlichia canis* (Ec), and *Leishmania infantum* (Li) and the *Dirofilaria immitis* (Di) antigen in dogs from different Spanish regions.

## Methods

### Bioclimatic characteristics of the study area

The survey was carried out in seven different eco-epidemiological regions of Spain. The Iberian Peninsula shows two main regions of flora and vegetation, the Mediterranean and Eurosiberian regions. This last region covers the north of Spain where climate and vegetation are typically oceanic, with warm summers and cool winters and rainfall evenly distributed all year round. The rest of the peninsula falls within the Mediterranean region. Here, summers are dry and hot, and most rainfall occurs in autumn and spring. Moreover, coastal areas show a milder, more humid climate, and mountain ranges have a shielding effect from an oceanic influence determining a climate of extremely cold winters and very hot summers.

### Veterinary clinics and dogs

The study was carried out in accordance with the International Guiding Principles for Biomedical Research Involving Animals, issued by the Council for the International Organizations of Medical Sciences. The owners of the dogs enrolled were previously informed about the study protocol.

**Figure 1:**
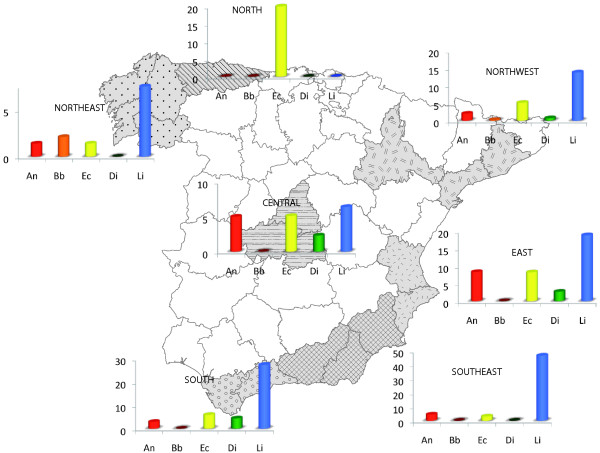
CVBD seropositivity recorded for seven Spanish geographical regions.

The dogs examined were 1100 owned dogs attending 57 veterinary clinics in central (187 dogs; 7 clinics), eastern (90 dogs; 5 clinics), southern (75 dogs; 5 clinics), southeastern (105 dogs; 5 clinics), northern (15 dogs; 1 clinic), northeastern (465 dogs; 22 clinics) and northwestern (163 dogs; 11 clinics) Spain (see Figure 1).

Dogs were subjected to the same protocol to compile a brief clinical record based on a questionnaire and physical examination. Blood samples were collected from all dogs. The data collected were correlated with age, sex, abode (indoors, outdoors or mixed), and the presence of clinical signs compatible with any CVBD. The dogs were 6 months to 18 years of age; 593 were male (489 entire, 104 neutered) and 507 female (405 entire, 102 neutered).

### Serologic testing

Anti-An, -Bb, and -Ec antibodies and the Di antigen were determined using the 4DX SNAP® Test (IDEXX Laboratories) and anti-*L. infantum* (Li) antibodies using the *Leishmania* SNAP® Test (IDEXX Laboratories) in blood and/or serum samples.

### Statistical analysis

Seropositivity rates were compared according to age, sex, abode and the presence of clinical signs. Associations between CVBD-agent seropositivity and the remaining variables were assessed using the chi-squared test. All statistical tests were performed using SPSS 19.0 software (SPSS Inc., Chicago, IL, USA). Significance was set at *p ≤ 0.05*.

## Results

Overall seropositivity rates for the five CVBD agents were: Li (15.7%), Ec (5%), An (3.1%), Di (1.25%) and Bb (0.4%). Rates obtained by geographic region are shown in Table 1. Seropositivity to the Bb antibody and Di antigen was similar in the seven regions. Seropositivity to An was significantly higher (8.3%) in the east than the remaining regions (0-5%), the rate for Ec was significantly higher in the north (20%) and that for Li was significantly higher in the south (46.6%) and southeast (27.4%). In the north of Spain, no dog tested was Li seropositive (Table 1).

**Table 1:** Seropositivity for the CVBD studied by geographical region

**Geographic region**		**CVBD**				
		**Positive/Total (%)**				
		**An**	**Bb**	**Ec**	**Di**	**Li**
**Central**	(n = 187)	9/180 (5)	0/176 (0)	9/176 (5.1)	4/176 (2.3)	10/157 (6.4)
**Northeast**	(n = 465)	9/451 (2)	1/451 (0.2)	23/451 (5.1)	3/451 (0.7)	60/434 (13.8)
**East**	(n = 90)	6/72 (8.3) *	0/73 (0)	6/73 (8.2)	2/73 (2.7)	13/69 (18.8)
**Southeast**	(n = 105)	2/45 (4.4)	0/30 (0)	1/33 (3)	0/31 (0)	41/88 (46.6) **
**South**	(n = 75)	2/68 (2.9)	0/68 (0)	4/68 (5.9)	3/68 (4.4)	20/73 (27.4) **
**Northwest**	(n = 163)	2/143 (1.4)	3/143 (2.1)	2/143 (1.4)	0/142 (0)	12/159 (7.5)
**North**	(n = 15)	0/15 (0)	0/15 (0)	3/15 (20) *	0/15 (0)	0/15 (0) **
**Total**	**(n = 1100)**	30/976 (3.1)	4/956 (0.4)	48/959 (5)	12/957 (1.25)	156/995 (15.7)
***p value***		*p = 0.04*	*p = 0.07*	*p = 0.04*	*p = 0.06*	*p < 0.001*

* *p < 0.05*; **p < 0.001. Abbreviations: An, *Anaplasma phagocytophilum/Anaplasma platys*; Bb, *Borrelia burgdorferi*; Ec, *Ehrlichia canis*; Di, *Dirofilaria immitis*; Li, *Leishmania infantum*.

No associations were observed between sex and any CVBD (*p ≥ 0.05*). Seropositivity towards Ec (40%) and Bb (6.7%) was higher in dogs under one year of age compared to adults (*p < 0.05*), while no differences in the rates recorded for An, Di or Li were detected between the two age groups.

Seropositivity to An and Li was significantly higher for dogs that lived outdoors compared to indoors. No link was detected between the seropositivity rate observed for Bb, Ec and Di, and place of abode.

When stratified by the presence or absence of clinical signs, percentages of An, Bb, Ec, Di and Li seropositive dogs were 2.1%, 0%, 1.7%, 0.5% and 4.2% for the subset of dogs with no clinical signs (n = 556), and 3.8%, 0.6%, 7.5%, 1.8% and 25.9% for those with clinical signs compatible with CVBD respectively (n = 507) (Table 2). The main clinical signs described by the veterinarians were: apathy, anorexia, anaemia, lymphadenomegaly, digestive disorders, skin lesions characterized by alopecia, seborrhoeic dermatitis, erythema, scaling and hyperkeratosis, as well as ulcerative lesions, and onychogryposis.

**Table 2:** Seropositivity for CVBD according to the epidemiological variables analyzed

**Epidemiological variable**		**CVBD**				
		**Positive/Total (%)**				
		**An**	**Bb**	**Ec**	**Di**	**Li**
**Age (years)**	<1	2/17 (11.8)	1/15 (6.7)**	6/15 (40)**	0/15 (0)	0/14 (0)
	1-3	12/369 (3.2)	1/361 (0.3)	7/362 (1.9)	2/361 (0.5)	60/370 (16.2)
	3-7	7/269 (2.6)	0/261 (0)	12/261 (4.6)	2/262 (0.8)	50/270 (18.5)
	>7	7/307 (2.3)	2/305 (0.6)	23/307 (7.5)	6/305 (2)	44/327 (13.5)
	Unknown	2/14 (14.3)	0/14 (0)	0/14 (0)	2/14 (14.3)	2/14 (14.3)
	***p value***	*p = 0.14*	*p = 0.001*	*p < 0.001*	*p = 0.3*	*p = 0.14*
**Sex**	Male	21/531 (4)	3/526 (0.6)	23/528 (4.4)	9/527 (1.7)	94/546 (17.2)
	Female	9/445 (2)	1/430 (0.2)	25/431 (5.8)	3/430 (0.7)	62/449 (13.8)
	***p value***	*p = 0.08*	*p = 0.16*	*p = 0.4*	*p = 0.14*	*p = 0.16*
**Clinical signs**	Asymptomatic	9/433 (2.1)	0/416 (0)	7/417 (1.7)	2/416 (0.5)	19/449 (4.2)
	Symptomatic	19/506 (3.8)	3/503 (0.6)	38/505 (7.5)**	9/504 (1.8)	133/513 (25.9)**
	Unknown	2/37 (5.4)	1/37 (2.7)	3/37 (8.1)	1/37 (2.7)	4/33 (12.1)
	***p value***	*p = 0.13*	*p = 0.11*	*p < 0.001*	*p = 0.07*	*p < 0.001*
**Place of abode**	Indoor	6/360 (1.7)	0/346 (0)	14/346 (4)	2/346 (0.6)	26/354 (7.3)
	Outdoor	18/357 (5)*	1/355 (0.3)	22/355 (6.2)	5/355 (1.4)	86/362 (23.8)**
	Mixed	5/256 (2)	3/252 (1.2)	12/255 (4.7)	5/253 (2)	43/276 (15.6)
	Unknown	1/3 (5.2)	0/3 (0)	0/3 (0)	0/3 (0)	1/3 (5.2)
	***p value***	*p = 0.01*	*p = 0.07*	*p = 0.23*	*p = 0.28*	*p < 0.001*

* *p < 0.05*; **p < 0.001. Abbreviations: An, *Anaplasma phagocytophilum/Anaplasma platys*; Bb, *Borrelia burgdorferi*; Ec, *Ehrlichia canis*; Di, *Dirofilaria immitis*; Li, *Leishmania infantum*.

In addition, seropositivity rates for Li and Ec were correlated with the presence of clinical signs in the dogs examined. No correlation was detected, however, between seropositivity for Bb, An and Di and the presence of clinical signs.

## Discussion

This study is the most complete survey of CVBD-agent seropositivity conducted in Spain. Most prior studies have been limited to a single region [21]. Overall, 37.1% of the dogs were seropositive for at least one of the five CVBD agents examined. The highest seropositivity rate detected was that of Li (15.7%) and the lowest Bb (0.4%), the seropositivity rate for each of these CVBD pathogens varying according to the geographic region. Despite differences detected in seropositivity to each CVBD-agent among each of the seven regions, the travel history of each dog was not included in the questionnaire, such that we cannot rule out the possibility that dogs were infected outside their home region [14].

The present study examines several epidemiological variables to assess possible associations with CVBD-agent seropositivity. No link was detected between sex and each CVBD (*p ≥ 0.05*) although seropositivity rates for Ec (40%) and Bb (6.7%) were higher in dogs under one year of age compared with adults (*p < 0.05*). These data suggest a need for further studies designed to determine the effects of age on CVBD-agent seropositivity since we only examined 17 young dogs.

No significant correlation was detected in our study between the presence of clinical signs in a dog and its positivity for Bb, An or Di. In contrast, Li or Ec positivity was correlated with the presence of clinical signs in the dogs examined (*p* < 0.05). This finding is consistent with reports indicating that clinical signs are commonly observed in dogs infected with *L. infantum* and *E. canis*[14,16,64,65].

CVBD have been correlated with the presence of vectors such that prevalences should be higher in dogs living outdoors due to their greater cumulative exposure to the agents these vectors transmit. However, we detected no association between positivity for Bb, Ec and Di and place of abode, though dogs living outside showed a higher rate of An and Li. Seropositivity towards An was significantly higher in dogs living outdoors, in agreement with data obtained in dog shelters in northwest (45.3%) and central (19%) Spain [20,21]. It would be interesting to collect information on whether the dogs were protected with an ectoparasiticidal agent since some of these insecticides are able to prevent CVBD [66-70]. Macrocyclic lactones have also been found to be effective against canine heartworm [46].

In Europe, *A. phagocytophilum* is transmitted by the tick *Ixodes ricinus*, whose distribution range is limited to areas of high humidity and cold temperatures, while *A. platys* could be transmitted by *Rhipicephalus sanguineus,* widely distributed across the Iberian Peninsula. Our results revealed a high seropositivity for this agent in eastern (8.3%) and central (5%) Spain, and a lower seropositivity in the north (0-2%). In prior studies conducted on dogs attending veterinary clinics in the northwest and east of Spain, similar rates of 5% and 11.5%, respectively, have been reported [19,20]. Despite the good sensitivity and specificity of the test used to detect antibodies against *Anaplasma* (99.1% and 100%, respectively), serological cross-reactivity between *A. phagocytophilum* and *A. platys* has been described in experimentally infected dogs [71]. Thus, PCR is needed to identify the *Anaplasma* species. Our results could therefore indicate exposure to the *Anaplasma* genus with no information provided at the species level. So far, *A. phagocytophilum* has not been isolated in Spanish dogs. It is likely that the antibodies detected in this study were anti-*A. platys* antibodies since we noted a higher seropositivity to An in areas where the presence of *R. sanguineus* is common. It is also true that *I. ricinus* is the common vector of *B*. *burgdorferi* and *A. phagocytophilum*, yet we found no An/Bb co-infections. However, dogs from some regions could be infected with *A. phagocytophilum* since this agent has been isolated from *Ixodes* ticks [72,73] and has also been detected in sheep, goats, cows, deer, birds [73-75] and even human beings [76,77].

Antibodies against Bb were only detected in four dogs, three in the northwest (2.1%) and one in the northeast (0.2%). The Snap 4DX kit only detects these antibodies during active infection [78] such that this could explain the low seroprevalence recorded. Other serological studies in which the Snap 3DX or 4DX methods were used have provided similar results [19,21]. Our data indicate significantly higher seropositivity for Bb in the younger dogs (<1 year), though this finding requires confirmation since our study only included 17 young dogs.

The bacterium Ec is transmitted by *R. sanguineus*. This tick is the most common tick found in dogs which explains the wide distribution of the disease [79]. *R. sanguineus* has been detected across Spain though we observed a significantly higher seropositivity to Ec in the north, where 6 out of 15 dogs were seropositive, while the overall prevalence of Ec was 5%. The low number of dogs surveyed precludes reliable estimates of the real prevalence of this disease. However, the higher seropositivity rate detected in the north could reflect the fact that *E. canis* infection is not limited to dogs, and that other wild canids (wolves, foxes, coyotes) may serve as reservoirs of infection [80]. Effectively, in northern Spain (Asturias), wild canids could live in close contact with domestic canids. There is also evidence that the prevalence of Ec is higher in rural areas or among stray dogs [20] than in urban areas [81]. Other authors have reported a similar Ec seroprevalence for different areas of the country in household dogs (3.13- 16.7%) [19,20] along with a higher seroprevalence (54.7%) in stray dogs from the northwest of Spain [20].

*D. immitis* antigen was detected in 1.25% of the dogs examined here. Higher rates were recorded for central (2.3%) and eastern (2.7%) regions of the country while Di antigen was not detected in dogs from the north and northwest. It is known that climate and environmental factors determine the geographical distribution of its vector (Culicidae), allowing it to complete its life cycle and consequently the life cycle of Di [42,82]. In Spain, similar surveys have identified Di in large areas of the country, often in irrigated zones [42]. A high prevalence of Di has been reported in the Canary Islands (19.2-67.02%)[38,40,41,83], Mediterranean coast (6.3-39%)[84] and southern regions (8.5-36.7%) [38,39]. At present, while in certain areas (e.g. Gran Canaria) prevalence is decreasing probably due to preventive measures [41,42], in other areas where Di was considered non-endemic (La Rioja and La Coruña), the first cases of canine dirofilariosis have been detected [43,44,85]. A possible explanation for the lower prevalences detected here is that the ELISA test can give rise to false negatives in dogs with low heartworm burdens or in blood samples from dogs infected only by male worms [86]. Besides, these types of study are difficult to compare due to differences in the diagnostic techniques used (PCR, agglutination test, ELISA, etc.), the size and origin of samples, and the study season.

As expected, seropositivity towards *L. infantum* was the highest of all the CVBD-agents analyzed (15.7%). Li antibodies were detected in all the geographic regions except the north, and higher rates were recorded for the south (27.4%) and southeast (46.6%). Our Li seropositivity map is similar to those emerging from other Spanish surveys [65,87,88]. Other studies conducted in Spain [50,89] have detected a higher seroprevalence in some northern areas. Such differences between studies could be attributable to the different population analyzed, the sampling season and the diagnostic technique used.

Among the dogs testing seropositive for Li, 85.2% (133/156) showed clinical signs of leishmaniosis in the physical exam performed by the veterinarians (Table 2). This can be explained by the fact that CanL is well known to Spanish practitioners despite the wide variety of clinical signs this disease can show [53,90]. In addition, Li seropositivity was significantly higher in the subset of dogs that lived outdoors compared to those living at home or even both indoors and outdoors. The explanation for this could lie in the increased exposure of dogs that spend more time outdoors to phlebotomines [65,88,91]. Arthropod vector distribution and density cause differences in the regional distribution of CVBD-agent seropositivity. To understand the potential role of the vector, knowledge of its environmental requirements is fundamental. There is indeed a need for research targeted at the prevention, diagnosis, treatment and prevention of CBVD. Information on the prevalence and geographical distribution of these infections is essential for planning control measures and their surveillance thereafter. This preliminary overview of the current situation in Spain requires further work to complete the prevalence map of agents causing CBVD in this country.

## Conclusion

The findings of this study reveal that dogs in Spain are at risk of acquiring any of the five CVBD examined (leishmaniosis, heartworm, ehrlichiosis, anaplasmosis and Lyme disease). They also indicate that veterinarians across Spain need to include these diseases in their differential diagnosis and recommend the use of repellents along with prophylactic measures to prevent disease transmission by arthropod vectors. In addition, greater involvement on the part of public health authorities is needed given that some of the CVBD detected can be transmitted to humans.

## Competing interests

The authors declare that they have no competing interests.

## Authors’ contributions

GM, AS and XR designed the survey, GM drafted the first version of the manuscript and finalized the manuscript. AM performed the statistical analysis of data, constructed the tables, drafted the first version of the manuscript and finalized the manuscript. RG prepared the figures and reviewed and finalized the manuscript. All authors read and approved the final version of the manuscript.
